# Continuous m-Health Data Authentication Using Wavelet Decomposition for Feature Extraction

**DOI:** 10.3390/s20195690

**Published:** 2020-10-06

**Authors:** Timibloudi Enamamu, Abayomi Otebolaku, Jims Marchang, Joy Dany

**Affiliations:** 1 Department of Computing, Sheffield Hallam University, Sheffield, S1 1WB, UK; a.otebolaku@shu.ac.uk (A.O.); jims.marchang@shu.ac.uk (J.M.); 2 Center for Cyber Security, Communications and Network Research (CSCAN), University of Plymouth, Plymouth, PL4 8AA, UK; dany.joy@plymouth.ac.uk

**Keywords:** smartphones, bioelectrical signals, biorthogonal wavelet, approximation coefficients, detail coefficient, wavelet transform, smartwatch, m-health monitoring

## Abstract

The World Health Organization (WHO) in 2016 considered m-health as: “the use of mobile wireless technologies including smart devices such as smartphones and smartwatches for public health”. WHO emphasizes the potential of this technology to increase its use in accessing health information and services as well as promoting positive changes in health behaviours and overall management of diseases. In this regard, the capability of smartphones and smartwatches for m-health monitoring through the collection of patient data remotely, has become an important component in m-health system. It is important that the integrity of the data collected is verified continuously through data authentication before storage. In this research work, we extracted heart rate variability (HRV) and decomposed the signals into sub-bands of detail and approximation coefficients. A comparison analysis is done after the classification of the extracted features to select the best sub-bands. An architectural framework and a used case for m-health data authentication is carried out using two sub-bands with the best performance from the HRV decomposition using 30 subjects’ data. The best sub-band achieved an equal error rate (EER) of 12.42%.

## 1. Introduction

The use of smartphones has increased over the years with many services adapting to mobile applications. The growth has seen competition in the use of mobile applications from marketing and advertising to goods and services. This has increased investment in the provision of services using apps on mobile devices. This is because it is more convenient to access the services on mobile devices compared to traditional computing sets. It is estimated that the worldwide usage of the most popular mobile device, smartphones, is expected to reach an estimated 3.8 billion and 40% of world population expected to own a smart phone by 2021 [1]. Smart devices are valuable devices not only because of their sophistication but also because they are used to store sensitive data like health, business, and financial information data [2]. Additionally, most social networking applications such as Facebook, Instagram, Snapchat, and WhatsApp can be accessed through these devices with internet connection. Therefore, it is expedient to secure these mobile devices to prevent access by an unauthorized user. Knowledge based authentication mechanisms have been a traditional way for authenticating a user’s access to a device. The use of knowledge-based authentication to secure mobile devices has been effective but has its own limitations because the data used is static in nature. The use of secret information known to the user can be forgotten or can be obtained by another person if written down [3] or someone can easily brute force especially when the passwords are short, or default settings are not changed. Also, most knowledge-based authentication requires periodic resetting. To improve on the limitation of knowledge base user authentication on a mobile device, biometric modalities is becoming prevalent, mainly because continuous form of user authentication can be in place in adopting such technique. Thus, recent research focuses more on the use of continuous and transparent biometric modalities for user authentication in smart mobile devices [4]. The adoption of easy and efficient way of extracting biometric data for implementing mobile user authentication is also improving because of interconnectivity of smart devices and their inbuilt sensors. Smartwatches are example of these devices that are becoming popular mainly because of the incorporation and integration of multiple sensors. The increase in their technological advancement has enhanced their functionalities and capabilities. For example, most smartwatches have the capability of accepting and declining calls, reading short message service (SMS), listening to music and use for navigation. Such devices also enable the user to monitor their health through activity related data from the sensors. Through these functionalities, smartwatches could be used to enhance the authentication technique of mobile devices. Although mobile user authentication implementation using smartwatch eliminates the issue of memorability however, the smartwatch can only authenticate the device when the user is near the smartwatch. So, brute-force attack and an attempt to get into the system by any other user will be very challenging because it will be extremely hard to replicate biometric data like bioelectrical signal. A smartwatch can extract bioelectrical signals, context awareness data and transmit it to a mobile device [5,6] for implementing mobile user authentication. As stated earlier, WHO emphasizes the potential of m-health technology by delivery of Cluster of Health Systems and Innovation [7]. Smart mobile device and its applications have been integrated into the health system with telemedicine and telehealth via the Internet of Things (IoT) [8]. In this paper, a biometric data (heart rate variability) is extracted using a smartwatch and transmitted to a smart phone. The extracted heart rate variability signal is decomposed using biorthogonal wavelet, a comparison analysis is done to identify the most suitable component of the signal to use for m-health data authentication for remote health care monitoring with a case study done.

## 2. State of the Art

**Figure 1:**
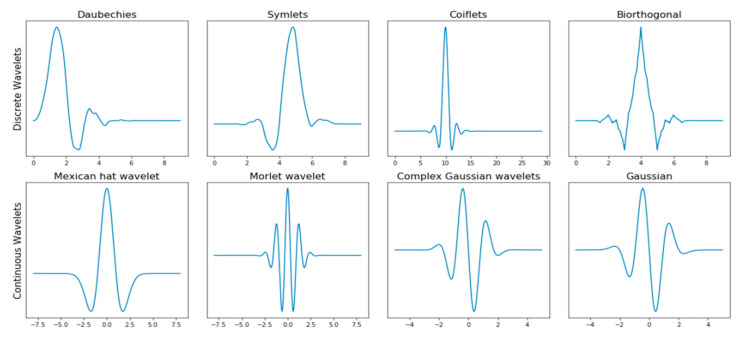
Wavelets families of discrete wavelets continuous wavelet.

Several techniques have been used for feature extraction including wavelet transformation. Wavelet transform is widely used for an extraction of non-stationary bioelectrical signal features [9]. This work used discrete wavelet transform (DWT) because it is popular for measurement and analysis of time-frequency and spectral component variation [10,11]. The method has been used extensively [12,13,14]. It enables the extraction of features that vary in time and is useful for analyzing transient signals [15,16]. There are several types of DWT which include, Morlet, Symlets, Mexican Hat, Haar, Daubechies, Coiflets, Meyer and Biorthogonal wavelets [17,18] as shown in Figure 1. Morlet wavelet function returns both amplitude and phase information for capturing oscillatory behaviors of a signal wavelet and it is common for the time–frequency analysis of acoustic signals [19,20]. Mexican hat wavelet transform has a more repeatable response for high frequency features and not a directional wavelet, mostly used for pointwise analysis [21,22]. Haar wavelet has the advantages of being simple and fast while dealing with memory efficiency, it can separate data classes without significantly losing the original data information [23]. Daubechies’s wavelets are endowed with symmetry with the energy spectrum concentrated around low frequencies and efficient for dimension reduction of image classification [24,25]. Symlets wavelets are nearly symmetrical wavelets with properties like Daubechies wavelet [26]. Coiflets apply the approximation properties depending on the number of vanishing wavelet moments [27]. Meyer wavelet has the advantage of better localization characteristics which are defined in the frequency domain of a signal while biorthogonal wavelet has linear phase filter banks with symmetric property, it is useful for signal and image reconstruction, it has an advantage of dual filter which corresponds to a fixed wavelet filter used for signal decomposition [28,29,30]. Bioelectrical signals extracted from smartwatch is expected to come with some level of noise, therefore using a wavelet with a filter bank to reduce the noise will be most suitable. Another advantage will be the decomposition of signal; this will enable extracting a sample of the signal for feature extraction. The biorthogonal wavelet family includes Bior1.1, 1.3, 1.5, Bior2.2, 2.4, 2.6, 2.8, Bior3.1, 3.3, 3.5, 3.7, 3.9, Bior4.4, Bior5.5 and Bior6.8. Biorthogonal wavelet transforms decompose a signal into approximation and detail coefficient. The Approximation and Detail Coefficient contains relevant information of a signal from which features can be extracted. Each n-level of the sub-band further decomposes the bioelectrical signal into a high and low frequency signal component [13].

**Figure 2:**
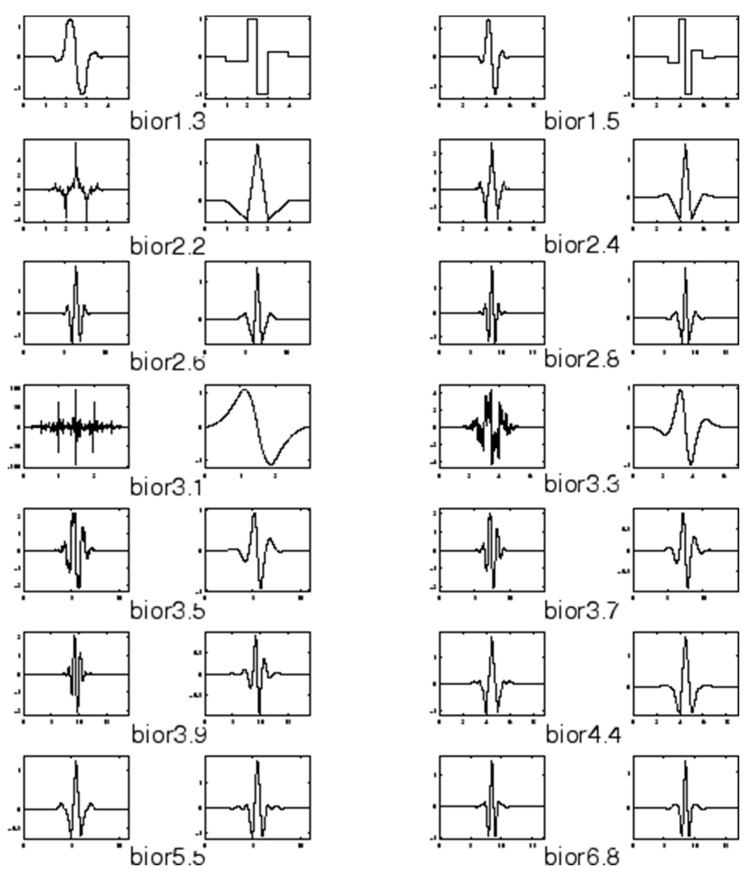
Showing the comparison of the decomposed signal of biorthogonal family.

The biorthogonal wavelet signal when reconstructed or decomposed as shown in Figure 2 differ in signal structure from each other which makes the properties different. Several literatures have shown that bioelectrical signals contain noise therefore it requires pre-processing. The application of biorthogonal wavelet filters some of the noise in the signals because biorthogonal wavelet uses a filter bank when decomposing the signals into sub-bands.

### 2.1. Biorthogonal Wavelets

One of the objectives of this work is focused on the performance of features extracted from biorthogonal wavelet decomposition by applying the most suitable sub-band of either approximation coefficient or detail coefficient for extracting features for classification. Majority of the prior work using biorthogonal wavelet [31,32,33,34,35,36,37] used either approximation coefficient or detail coefficient mostly from signals extracted using an intrusive method. The use of smartwatch reduces the intrusiveness in the extraction of the signal; therefore, a comparison analysis is to determine the most efficient biorthogonal wavelet family for extracting features. The most efficient coefficient is then used to implement a patient’s data authentication before it is stored. It is important to note that mobile health (m-health) is on the rise and useful for daily life related health monitoring of patients; however, most of the emphasis is on the usability and availability of the health data [7,38]. The authenticity of the data should be continuously monitored too to establish trust. This work proposed a framework design for implementing a patient m-health data authentication for e-health monitoring system. The framework includes transparent data extraction, pre-processing, feature extraction and classification for authentication of a patient’s data.

### 2.2. Heart Rate Variability

There are several works that have used heart rate variability (HRV) for authentication. [39] used a wireless device from Alive Technologies to extract HRV for authentication using 15 subjects. The device is connected to specific part of the body and with 2 sets of scenarios for the data collection. In another work using HRV for securing body sensor network (BSN), [40,41] designed a textile electrode to extract HRV using 24 males for the experiment. The experiment like the first set a scenario for the participants too. The used of a device that are not compatible with everyday use will comes with some limitation for health monitoring propose. This work extracts the HRV without a scenario set for the participants and used a smartwatch which is convenient for wellbeing monitoring. The use of wearables for monitoring patients’ health conditions remotely is increasing because of improvement in technology. These devices include smartwatches which should meet some requirements. These requirements include availability of the required data at any time, data privacy and security, usability of the data and accuracy of the data. Heart rate variability (HRV) is extracted from the successive heartbeats R-R interval. This has a relationship with the electrocardiography (ECG) because R is the peak of the QRS complex used for calculating the ECG. ECG has been used for identification of human with good accuracy [42,43]. There are more smartwatches in the market today capable of extracting heart rate variability signal compared to years back, therefore this work used one of these smartwatches for this experiment.

## 3. Methodology

In this section, the experimentation and the method followed is discussed in detail with several experiments carried out. The first experiment used 12 subjects to select the most suitable features to use for the second experiment. The data for the first experiment used heart rate variability signal extracted for 240 s. The other experiments also extracted the heart rate variability signal for 7200 s for each of the 30 subjects.

### 3.1. Transparent Data Extraction

**Figure 3:**
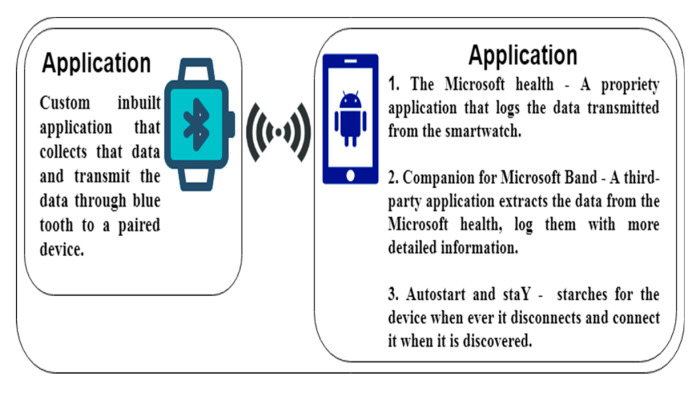
A high-level view of the system used, comprising a smartwatch and smartwatch.

**Figure 4:**
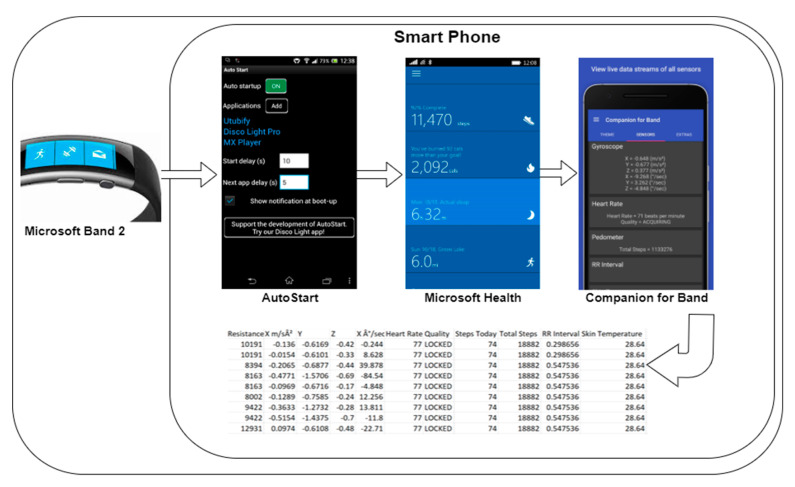
Illustration of the mobile application process within the smartphone.

To apply a robust dataset, 30 subjects are used for the evaluation of the statistical features extracted and the comparison experiment is done using different biorthogonal wavelet family. Using a smartwatch, heart rate variability signals are extracted for 4 h without a specific task. The data is transmitted to a smart phone for storage via a Bluetooth connection. A sampling rate of 8 samples per second is used to extract enough data points per second. Figure 3 shows the applications used for the extraction of data from the smartwatch to the smartphone. The different android applications in the phone perform different functions to continuously extract the data if the two devices are within a communication distance. Within the smart phone as shown in Figure 4, the AutoStart and StaY application searches for the smartwatch to pair with the smartphone through Bluetooth and reconnects it whenever it disconnects. This enables the smartwatch and smartphone reconnect when either the smartwatch or smartphone is switched on. The Microsoft health is a proprietary application customized to communicate with the inbuilt application on the smartwatch, i.e., when the connection is established, the Microsoft health application extracts the data and transfers it to the Companion for Band application, this enable the information to be logged in a better and more presentable format. The combination of the three application on the smartphone enable a seamless data extraction from the smartwatch.

### 3.2. Data Segmentation for Continuous Authentication

The segmentation of the data is important for the implementation of a continuous user authentication. This is the division of the signal into smaller sets of signals for feature extraction, it is also important because the sets of smaller signals should be rich enough for useful features extraction. The data is expected to be extracted within a time frame for processing. The interval needs to extract enough data for feature extraction is significant for effective and continuous authentication of the data before it is stored. A faster authentication process is desired therefore, a frame of 3 s window data extraction is chosen for the work based on prior work [44]. Table 1 shows the data segmentation in 3 s.

### 3.3. Biorthogonal Wavelet Decomposition

The signal processing involves the reduction of the noise using filters. Biorthogonal wavelet processes a signal using low pass (L) and high pass (H) filters. The output is either a decomposition or reconstruction of the signal of the low pass or high pass filters and it is categorized into four different outputs:

- Low-pass decomposition filter = Lo_D
- High-pass decomposition filter = Hi_D
- Low-pass reconstruction filter = Lo_R
- High-pass reconstruction filter = Hi_R

This work applies the decomposition of the signal using the high and low pass filters to the n number of signal samples generated. The wavelet decomposition applies a low-pass filter and high-pass filter depending on the cut off frequency of the approximation and detailed coefficient. The amount of vanishing moment is determined by the coefficients h[n] of the filter h. The greater the number of vanishing moments, the better the reconstruction of the signal to accurately represent unique feature points. Biorthogonal filters are symmetric with one to three vanishing moments with a certain degree of varying the coefficient without destroying the vanishing moment properties [45].

### 3.4. Feature Extraction

Based upon prior works on bioelectrical feature extraction, statistical feature has been employed effectively for transient signals [4,13,46]. An initial 12 subjects are used to test run 13 statistical features using the first level of sub-bands decomposition. The initial 13 features used in the first experiment are defined below:

1. Variance: This is the sum of square distance of the bioelectrical signal.
  (1) $$Variance=\;\frac{\sum {\left(X-\mu \right)}^{2}}{N}$$
2. Mean of the energy: The signal mean of the energy is the energy average value of the bioelectrical signal.
  (2) $$Mean\;of\;the\;Energy=\frac{\sum_{r}\exp\left(-\beta E_{r}\right)E_{r}}{\sum_{r}\exp\left(-\beta E_{r}\right)}$$
3. Minimum energy: This is the lowest energy value of the bioelectrical signal
  (3) Minimum Energy=Minimum Signal Energy
4. Maximum energy: This is the highest energy value of the bioelectrical signal.
  (4) Maximum Energy=Maximum Signal Energy
5. Mean: These are the values diversity of the data around the median.
  (5) $$Mean=\;\frac{1}{n}\overset{n}{\underset{i=1}{\sum }}x_{i}$$
6. Minimum amplitude: This is the lowest point from the equilibrium point of the bioelectrical signal.
  (6) Min. Amplitude=Minimum displacement
7. Standard deviation (STD): This is the square root of the variance of a random variation.
  (7) $$STD=\sqrt{\frac{1}{n}\overset{n}{\underset{i=1}{\sum }}\;{\left(x_{i}-\mu \right)}^{2}}$$
8. Maximum amplitude: This is the highest point from the equilibrium point of the bioelectrical signal.
  (8) Max. Amp.=Maximum displacement
9. Range: This is the difference between the highest signal value and the lowest signal value.
  (9) Range=Maximum signal−minimum signal
10. Peak2peak: This is the difference between the maximum and minimum values of the bioelectrical signal.
  (10) P2P=Signal Maxi to Min diff. displacement
11. Root mean square (RMS): The RMS is the measurement of the magnitude of a set values within the signal.
  (11) $$Root\;Mean\;Square\;=\sqrt{\frac{1}{n}\sum_{i=1}^{N}X_{I}^{2}}$$
12. Peak magnitude to RMS ratio: This is the ratio of the largest absolute value of a signal to the root mean square (RMS) value of that signal.
  (12) $$Peak\;Magnitude\;to\;RMS=\sqrt{\frac{X_{\infty }}{\frac{1}{N}\sum_{n=1}^{N}X_{n}^{2}}}$$
13. Average frequency: this the arithmetic mean of the signal frequency.
  (13) $$Average\;Frequency=\;\frac{X_{1}+X_{2}\;+\;X_{3}\ldots X_{n}}{N}$$

**Figure 5:**
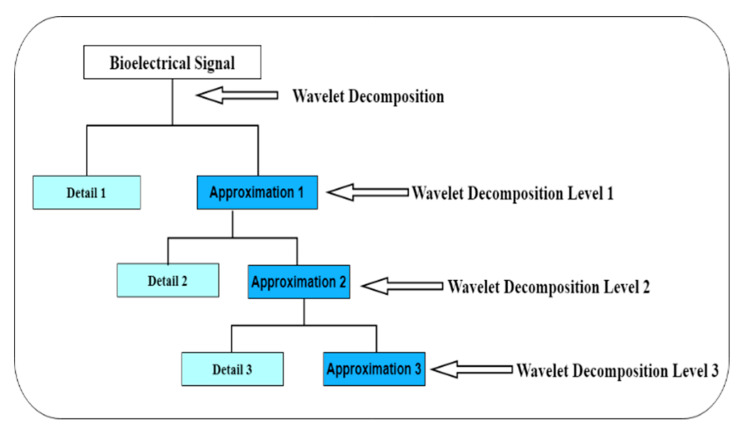
Wavelets decomposition of the detail and approximation coefficient.

**Figure 6:**
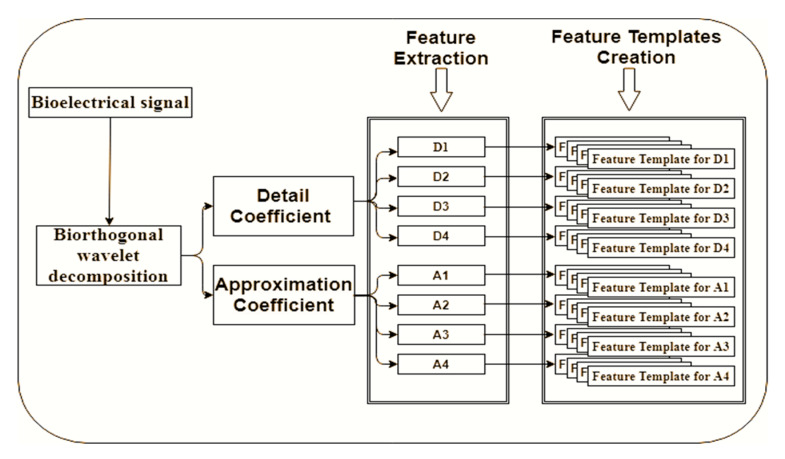
Wavelets schematic illustration of the feature extraction process.

From the 13 statistical features defined above, an evaluation of the features is done to select the best features for the other experiment. The final experiment used 30 subjects and applied the selected statistical features for level 1 to 3 sub-bands of detail and approximation coefficient decompositions as shown in Figure 5. The 13 statistical features for the initial experiment are extracted from the detailed $\left(D_{i}\right)$ and approximation $\left(A_{i}\right)$ coefficient. The features are extracted from each level of the decomposed signal of detail (D1–D3) and approximation (A1–A3) coefficient as shown in Figure 6.

### 3.5. Classification

**Figure 7:**
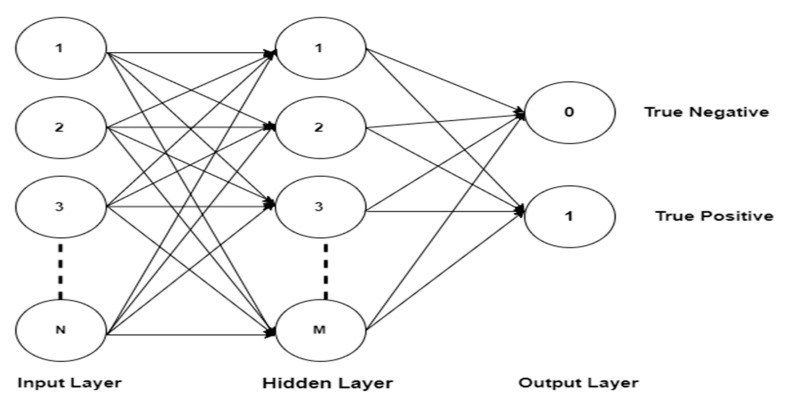
Neural Network Feedforward structure model.

Feed Forward Neural Network (FF-NN) is used for the classification of the 3 sub-band levels and the data authentication experiment. Neural network is one of the most widely used classifiers in bioelectrical signal classification as shown in prior works [13,44]. The feedforward neural network is basic among neural network for classification. FF-NN is used because it is the simplest for processing the neurons [47], it can easily map a set of input signals using the similarities to identify signal samples easily [41]. The use of mobile device for processing the data limit’s the computational power for large data processing therefore, FF-NN has advantage of modelling the part that cannot be modelled. It is also fault tolerant and can learn from noisy data [48] like the heart rate variability used in this work. The classification evaluation metric calculates the equal error rate (EER) using false acceptance rate (FAR) and false rejection rate (FRR). The EER is the point at which the FAR and FRR meet. The features extracted from the level 1 to 3 sub-bands of the decomposed signals are classified from each of the biorthogonal wavelet family. The (N) features are sent as input with 75 (M) nodes in the hidden layers used for the classification. The output is either true negative (0) or true positive (1) as shown in Figure 7, while the true positive indicates as the right patient data while true negative indicates as non-patient data.

### 3.6. Experimentation

The different experimentations of this work applied the same data pre-processing and segmentation, feature extraction and classification technique as earlier presented. The experimentation is divided into four:

- Experiment 1: the experimentation to select the most suitable features for use in the other experiments. The experiment used 12 subjects’ data for the 13 statistical features extraction experimentation.
- Experiment 2: the second experimentation is the classification of the sub-bands after extracting features. The 30 subjects’ data are used with 12 selected features for each sub-band for both the approximation of coefficient and detail coefficient for fifteen biorthogonal wavelet family.
- Experiment 3: this experiment is done on the fusion of the sub-bands. This experiment also used 30 subjects’ data with the 12 selected features for fusion of all the sub-band for both the approximation of coefficient and detail coefficient classification.
- Used case experiment: this is the used case data authentication using 30 subjects’ data.

## 4. Results

### 4.1. Result on Experiment 1: Selection of Statistical Features

**Figure 8:**
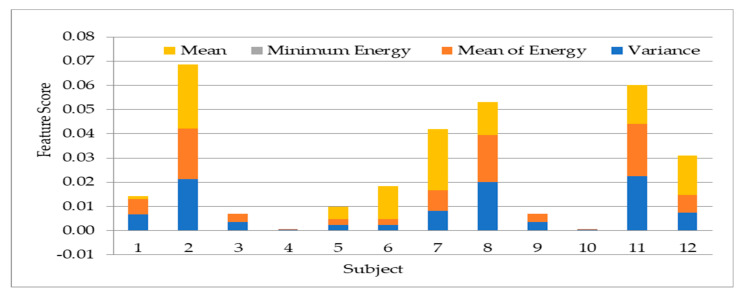
Variation of mean, minimum energy, mean of energy and variance on twelve subjects.

**Figure 9:**
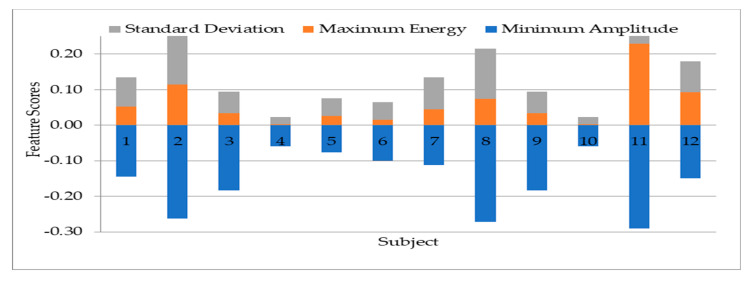
Variation of minimum amplitude, maximum energy, and standard deviation on twelve subjects.

**Figure 10:**
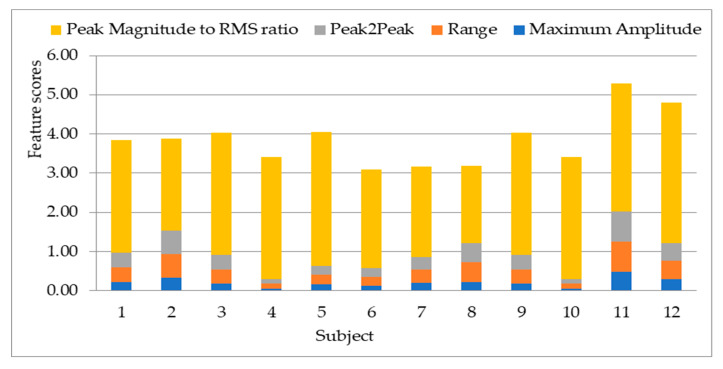
Variation of maximum amplitude, range, peak2peak and peak magnitude to root mean square (RMS) ratio on twelve subjects.

**Figure 11:**
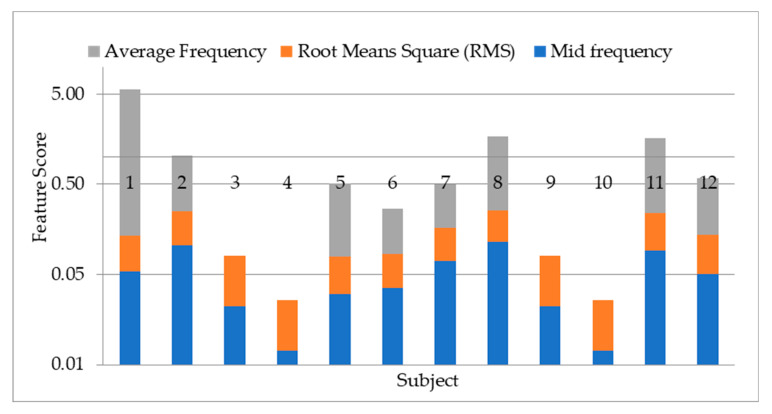
Showing variation of mid frequency, root mean square and average frequency on twelve subjects.

To study the discrepancy between the 12 subjects’ data used, the 13 statistical features are extracted from a segment of the bioelectrical signal. The output is tabulated to show the variations of the features across subjects. The feature variations are important in choosing the most effective features to apply on the biorthogonal wavelet sub-bands decomposition. The features are extracted with MATLAB using the first level detail coefficient of biorthogonal wavelet transform sub-band. The graphical representations of the extracted features in Figure 8, Figure 9, Figure 10 and Figure 11 is represented with different feature score ranges. The feature variations are important in choosing the most effective features for classification of subjects. The x-axis shows the different subjects while the y-axis shows the features scores. The disparity of score between the subjects shows good discriminatory information in the features. For example, Figure 8 shows the data for subject 11 and 12 having different score of the variance, mean of energy and mean for each subject with their feature scores are also different too. This is important as it is used to differentiate each subjects’ data because of the different information provided by the features associated with each subjects’ data. Figure 9 values range from 0.0 to 0.03 with the mean having the highest value. The graphical representation shows that the variation of variance and mean of the energy provides good value to discriminate subjects with minimum energy not having any value. The means has value for some scores but not all the subjects. Therefore, the minimum energy and the mean will not be ideal for use in classification of subjects. The variation of subjects by the minimum amplitude, maximum energy, and standard deviation as illustrated in Figure 9 with values ranging from −0.29 to 0.23 provides for useful discrimination, therefore the three will be selected for further feature extraction. Figure 10 shows that the maximum amplitude, range, peak2peak and peak magnitude shows a good discrimination across all the subjects. The features scores between the range and peak2peak are almost the same in some subjects’ data but will add value to the discrimination of the data; therefore, all the features are used. The variations of all features in Figure 11 except the average frequency have good values for discrimination. The mid frequency and root mean square will be selected while the average frequency will not be used for the experiment using 30 subjects’ data.

From the 13 statistical features, 12 statistical features of the mean, mean of energy, variance, minimum amplitude, maximum energy, standard deviation, maximum amplitude, range, peak2peak, peak magnitude to RMS ratio, mid frequency and root mean square were chosen. The 12 selected features are applied for the experiment using 30 subjects’ data. The features will be extracted from each of the decomposed signal of detail (D1–D3) and approximation (A1–A3) of the different biorthogonal wavelet family.

### 4.2. Result on Experiment 2: Classification Sub-Band Features

To extract features from the signal, fifteen different wavelets from biorthogonal wavelet family are used to decompose the signal into three sub-bands. To evaluate the performance of the three sub-bands of approximation coefficient and detail coefficient, the ten features selected are applied to three sub-bands of approximation of coefficient and detail coefficient from the data of 30 subjects. The use of 3 sub-band (decomposition level) is because of the bioelectrical signal frequency of the heart rate variability. This is necessary because it should correlate with the frequencies necessary for classification [48]. Neural network classifiers are used with the same number of neurons across all the sub-bands. The network size is not the best that can be applied but for equal evaluation across the sub-bands, network size 20 is used. Table 2 shows the output result for the sub-bands across approximation of coefficient and detail coefficient.

**Figure 12:**
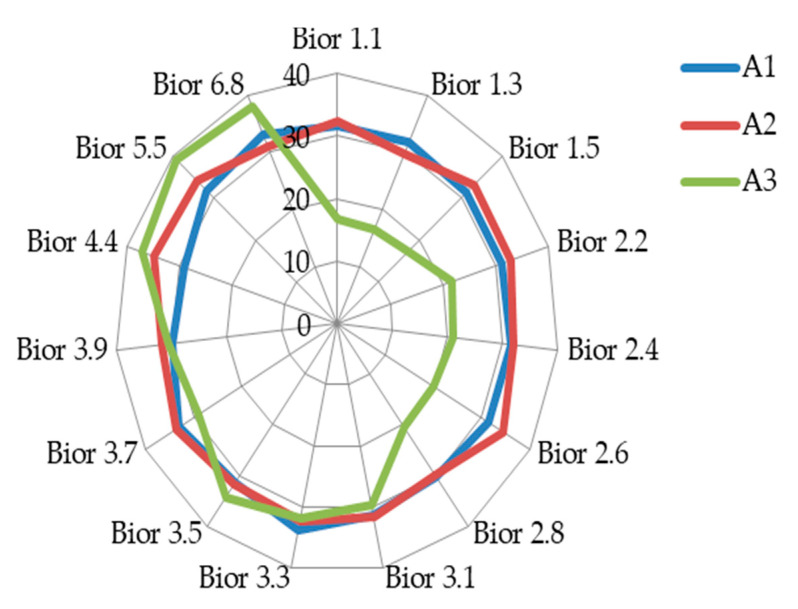
Showing classification accuracy comparison in equal error rate (EER) across various biorthogonal decomposition of approximation of coefficient features (A1: approximation of coefficient sub-band1; A2: approximation of coefficient sub-band 2; A3: approximation of coefficient sub-b).

From the three sub-band levels of approximation coefficient as illustrated in Figure 12, it is of interest to note that the sub-band 1 of the approximation coefficient seem to be consistent and within the region of EER of 30% to 35% except in bior4.4 where it is below EER of 30%. This implies that using the bior4.4 will produce almost the same result irrespective of the biorthogonal wavelet family used if it is suitable for the feature extraction of the dataset.

**Figure 13:**
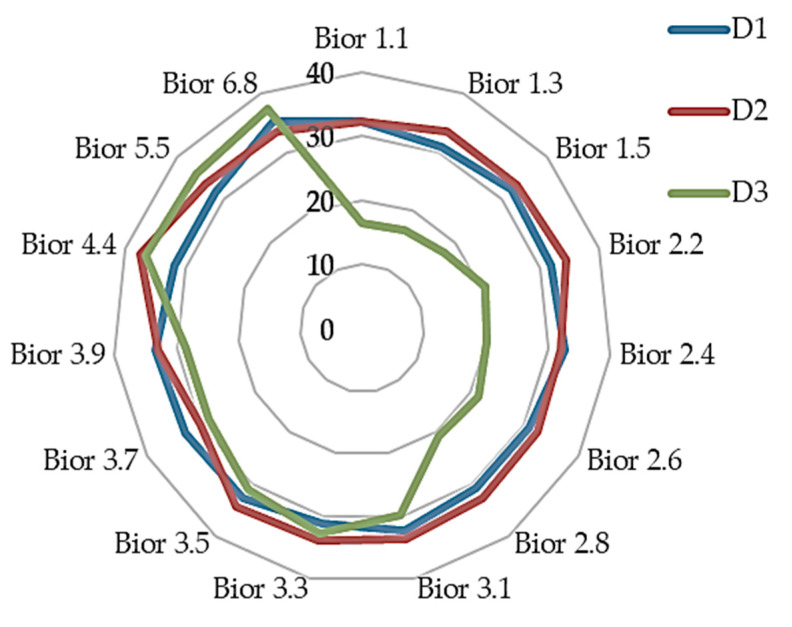
Showing classification accuracy comparison across various biorthogonal decomposition detail coefficient features (D1: detail coefficient sub-band1, D2: detail coefficient sub-band 2, D3: detail coefficient sub-band 3).

To further analyzes and compare the results, two sets of radar chart diagrams are used to show the fifteen biorthogonal wavelet family. Figure 12 shows detail comparison of the classification results using the fifteen biorthogonal wavelet decomposition of approximation between sub-band 1, 2 and 3. The results from the two classifications (Figure 12 and Figure 13) produced identical results for the approximation of coefficient and detail coefficient. However, a closer look at the radar chat show the Approximation of coefficient and detail coefficient sub-band results of sub-band 2 and 3 fluttered with in the EER of 30% to 35%. The 3 sub-band showed better performance in the first 7 biorthogonal wavelet wavelength for both the approximation of coefficient and detail coefficient.

The detail coefficient features of sub-band 1(D1) in Figure 13 shows the same pattern at the approximation of coefficient. The approximation coefficient and detail coefficient result of sub-band 2 followed the same pattern of sub-band 1 approximation with result within the EER region of 30% to 35%. The sub-band 3 of the approximation coefficient and detail coefficient has an interesting trend. The best results are from bior1.1 with EER of 16.66 to bior2.8 have an EER of 20.44% with a sharp gradient to a higher EER of 29.70% at bior3.1. The interesting phenomenon is the performance of approximation of coefficient from bior4.4 to bior6.8 where the EER is highest (among all the biorthogonal wavelet classification) compared to its performance from bior1.1 to bior2.8. This trend is notice in both diagrams.

### 4.3. Result on Experiment 3: Fusion of Sub-Band Features

**Figure 14:**
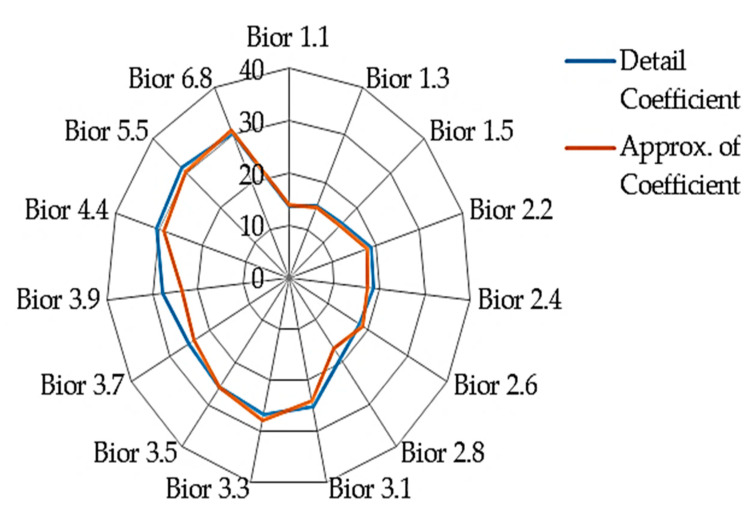
Comparison of the performance of detail and approximation coefficient feature classification.

Feature fusion is the combination of the extracted features together before classification. The application of fusion in biometric authentication enhances the performance therefore the fusion of the sub-band is initiated to improve its performance. The fusion is carried out by first extracting the features before fusing the three sub-band features for classification. The classification result in EER is shown in Figure 14. The approximation coefficient has shown better performance in all the classified results except on the bior3.3 with an EER of 26.7%. Table 3 shows the best four performing biorthogonal wavelets. The best result among them is the detail coefficient fusion of the bior1.1 three sub-band features scoring an EER of 13.80%. This is followed by the approximation coefficient fusion of the bior1.1 scoring EER of 14%.

## 5. Use Case and Evaluation

**Figure 15:**
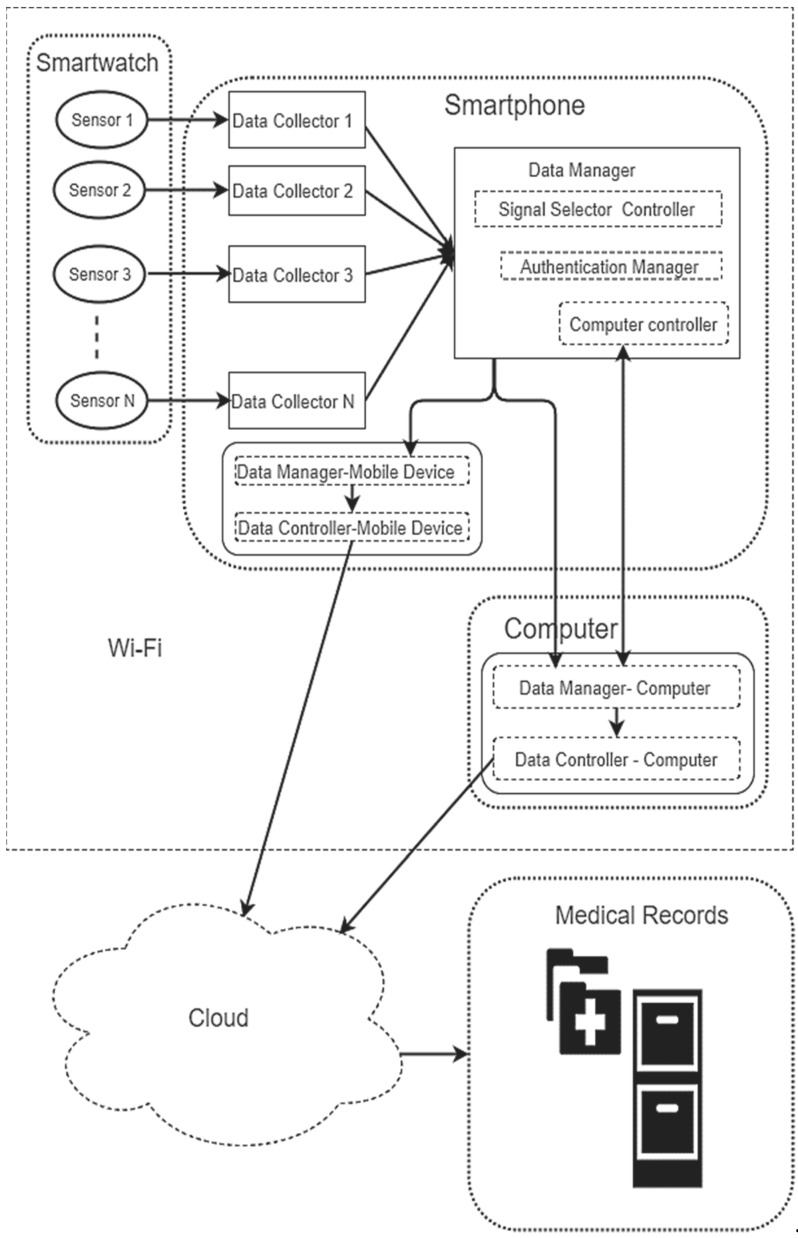
Showing proposed m-health data authentication framework.

The authentication of mobile healthcare data is of great concern to the health sector [49] therefore, presenting a framework to increase trust of healthcare data transmitted form wearable is necessary. The process described in this section includes an architecture of a use case, an authentication of the data from 30 subjects presented as patient’s health data and evaluation of the used case. The use case architecture as shown in Figure 15, will extract data from the patient using the smartwatch then transmit either through a Smart mobile device or computing system to the health care facility.

### 5.1. Use Case Architecture

**Figure 16:**
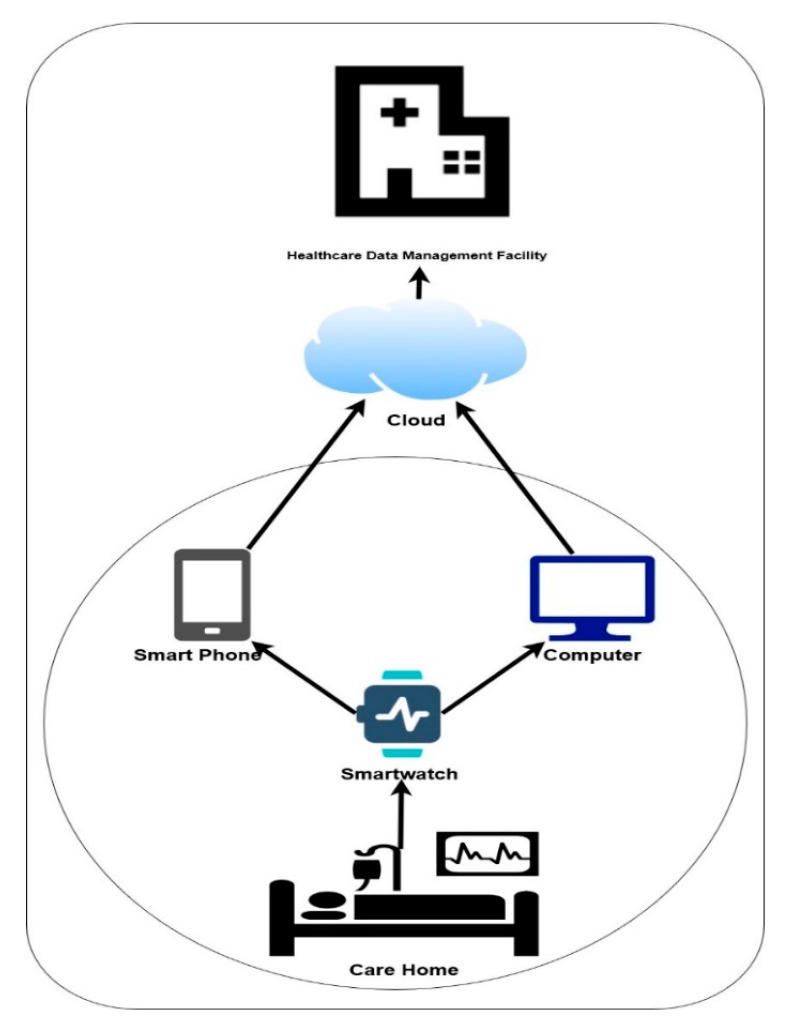
Use case schematic architecture.

Figure 16 shows a schematic architecture of the proposed used case system. The framework comprises different components which includes the following smartwatch, smartphone, computing system and healthcare data management facility.

#### 5.1.1. The Smartwatch

The smartwatch provides the technology to keep track of physical activity using sensors for tracking health information like heart rate variability, blood pressure and other activities useful for m-health monitoring [46]. The smartwatch is the primary data extractor for processing on either the phone or computing system in the framework.

#### 5.1.2. The Smartphone

Smartphone capability is on the increase, this includes its application for a variety of activities including data collection and processing [13]. The mobile devices in the architecture include the data collection, processing for transmission to healthcare data facility via the cloud. It also includes a manager for transferring data to the computing system.

#### 5.1.3. The Smartphone

The computing system provide a platform for processing the data as a compliment to the mobile. The computing system must be connected wirelessly to communicate with the smartwatch.

#### 5.1.4. Healthcare Data Management Facility

The healthcare data management facility maintains and collate data from patients that has been authenticated.

### 5.2. Data Authentication

The data authentication is done using 30 subjects (representing patients) with the data extracted for 14,400 s while going about daily active as usual. The data is extracted using the same procedure as describe earlier. The smartwatch is worn be the subject with the data extracted transparently and transmitted to a smart device. The data is taken from the smart device for processing. As stated earlier, this work is focusing on the extraction process and the processing of the data for authentication that the data is coming from the intended patient. The two sub-band fusion with the best performance is use for the verification process. The two datasets are used to take care of overfitting. The issue of noise in overfitting is dealt with by using decomposition of the signal with wavelets transform in the processing of the data therefore, using more datasets to overcome overfitting is taken care of.

**Figure 17:**
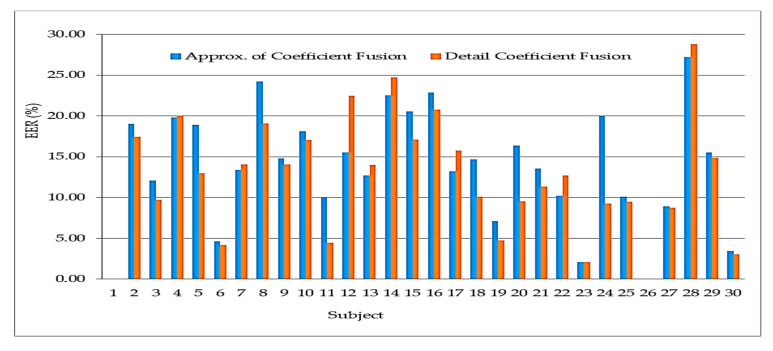
Result showing the verification of the data from 30 subjects using fusion of approximation and detail coefficient sub-bands.

The combination of the sub-bands is based on the preliminary results on the fusion of sub-bands. Findings from the fusion of features to discriminate between subjects has a positive outcome. It is interesting to note that the used case experiment using 30 subjects indicated the same trend in terms of the difference between the two best results. The initial experimental result had Detail Coefficient surpassing Approximation Coefficient with 0.2% EER while the verification experiment has the Detail Coefficient scoring the best but with a wider margin of 0.75% EER. More interesting is the fact that most subjects’ performance is directly related to the two results as shown in Table 4. For example, subject 1 and 26 have the same results (0% EER) on both results. The same is for subject 23 scoring 2.08% EER. The difference between the two classification results on individual assessment shows subjects 4, 6, 7, 9, 25, 27, 29 and 30 are close in terms of their EER as illustrated in Figure 17. The result shows the most appropriate biorthogonal wavelet decomposition to use for the verification is the detail coefficient.

### 5.3. Use Case Evaluation

The data authentication is evaluated using the classification dataset. The dataset is divided into training and testing. The testing dataset is for evaluating the performance of the proposal. Subject 1–10 is used as the patient while the remaining 20 subjects are used as non-patient. To authenticate the patient, the test data of each of the 10 patients is used against the test data of the 20 non-patients. Each of the 10 patients are classified 10 times therefore, the number of attempts to authenticate the 10 patents is 100 times while the 20 non-patients were used 2000 times. Table 5 shows the number of patients and non-patients accepted and rejected, and the success rate of the use case evaluation. A threshold of 9%, 10%, 11% and 12% EER is used to verify the patients. These thresholds are used as not to go above the best performing EER of the data authentication. To calculate the Success Rate the following formula is used:

(14) $$Sucess\;Rate\;for\;Patient=\left(\frac{Number\;of\;Patient\;rejected\;}{All\;Attempt\;to\;authenticate\;the\;Patient\;}\right)\;\times \;100$$

Equation (14) Success Rate for patient.

(15) $$Sucess\;Rate\;for\;Non-Patient=\left(\frac{Number\;of\;Non-Patient\;accepted}{All\;Attempt\;of\;using\;Non-Patient\;to\;authenticate}\right)\;\times \;100$$

Equation (15) Success Rate for non-patient.

### 5.4. Discussion

As earlier stated, the bioelectrical signal frequency determines the sub-band level to use. It is also necessary to note that different signals will ideally be used for different biorthogonal (bior) family for feature extraction if biorthogonal wavelet is used. To effectively discuss the results, the biorthogonal wavelet is divided into two regions based on the finding’s performance. The first region is from bior1.1 to 2.8 and the second from bior3.2 to bior6.8. The first region (bior1.1 to bior2.8) approximation of coefficient and detail coefficient shows that the sub-band 3 performed better than the sub-band 1 and 2 as illustrated in Figure 6 and Figure 7. The approximation of coefficient sub-band 1(A1) has a stable result in the region. This implies that irrespective of the biorthogonal wavelet family used within the two regions, the result is expected to be slightly different from each other except on bior4.4 where the finding has a better result, scoring below EER of 30%. Therefore, it will be most suitable to use bior4.4 as shown in the result. In general, sub-band (decomposition level) 1 of approximation of coefficient and detail coefficient of biorthogonal wavelet is suitable for bioelectrical feature extraction with the same frequency range of heart rate variability. To limit it to a region it will be most suitable to use sub-band 1 of bior1.1 to bior2.8 (first region) of approximation of coefficient and detail coefficient for feature extraction. The fusion of the sub-band improves the result of classification. Therefore, where it is necessary to apply fusion of the sub-bands considering the processing capacity of smartphones, the fusion of either bior1.1 or bior1.3 will be most desirable. The result in Table 3 shows sub-band 3 of detail coefficient using bior1.3 have the best result of EER of 16.41% and for the fusion of features, the best result is the fusion of detail coefficient scoring EER of 13.80%. The results from the thirty subjects have shown consistency with the earlier experiment showing the fusion of the detail coefficient of bior1.1 is effective in the classification of bioelectrical signals. The evaluation shows that a non-patient will most likely not be authenticated to be a patient.

## 6. Conclusions and Future Work

This paper investigated the authentication of m-health data for remote health care monitoring. The method used smartwatch and mobile device to extract the m-health data. To effectively evaluation the proposal, a case study is implemented with 30 subjects for the user authentication using a bioelectrical signal of heart rate variability. The work described how bioelectrical signals, in this case, the heart rate variability is processed using a portion of the signal after decomposition to implement data authentication before the health data can be stored. The experimental result for the used case shows the patient’s data is authenticated to 84% success rate while the non-patient is at 0.4% success rate.

One of the issues identified for future work is the fusion of more than one bioelectrical signal to improve the success rate. For health data collected remotely, a 100% success rate will be ideal. The more useful information is fused, the better the success rate. We would like to collect more data from additional sensors with dynamic selection of suitable features. We then hope to experiment with various machine learning algorithms including deep neural network algorithm.

## Figures and Tables

**Table 1:** Data segmentation for continuous data authentication.

Heart Rate Variability Segmentation Using 3 Sec.			
Types	Exp. 1	Exp. 2–3	Used Case Exp
Number of subjects used	12	30	30
Sampling rate	8	8	8
Data points per segment	24	24	24
Num. of Feature Segments of each subject	150	300	600
Number recorded time per subject in Seconds	3600	7200	14,400
Total number recorded time for all subjects in Seconds	108,000	216,000	432,000

**Table 2:** The three sub-band levels of approximation of coefficient and detail coefficient (A1: approximation of coefficient sub-band1; A2: approximation of coefficient sub-band 2; A3: detail coefficient sub-band 3; D1: detail coefficient sub-band1, D2: detail).

No	Wavelet Family	EER of Approximation of Coefficient and Detail Coefficient Classification Comparison in EER (%)					
		D1	A1	D2	A2	D3	A3
1	Bior1.1	32.27	31.54	32.21	32.31	16.41	16.66
2	Bior1.3	31.02	31.77	33.51	29.80	16.79	16.56
3	Bior1.5	32.33	31.42	33.32	33.21	17.56	17.20
4	Bior2.2	31.79	31.12	34.50	32.99	20.69	21.69
5	Bior2.4	32.59	31.55	32.06	31.87	20.02	20.95
6	Bior2.6	30.87	31.44	32.32	34.55	21.47	19.94
7	Bior2.8	30.83	30.39	32.66	29.81	20.79	20.44
8	Bior3.1	32.16	31.40	33.67	31.58	29.82	29.70
9	Bior3.3	31.10	33.64	33.74	32.18	32.82	31.90
10	Bior3.5	32.65	31.40	34.44	31.63	31.11	34.32
11	Bior3.7	32.73	32.90	30.08	33.67	28.52	28.95
12	Bior3.9	33.41	29.99	33.10	31.81	28.50	30.67
13	Bior4.4	31.85	29.03	37.57	34.86	36.54	37.16
14	Bior5.5	31.61	31.87	33.67	34.05	35.93	39.01
15	Bior6.8	35.22	33.07	33.59	30.94	37.40	37.90

**Table 3:** Showing the approximation of coefficient and detail coefficient classification.

Approximation and Detail Coefficient Classification Comparison in EER (%)		
Feature	Bior1.1	Bior1.3
Approximation of Coefficient Sub-band 3	16.66	16.56
Detail Coefficient Sub-band 3	16.41	16.56
Approximation of Coefficient Fusion	14.00	14.83
Detail Coefficient Fusion	13.80	14.89

**Table 4:** Showing feature fusion classification comparison in ERR (%).

Sub.	AF	DF	Sub.	AF	DF
1	0.00	0.00	16	22.84	20.76
2	19.04	17.46	17	13.22	15.73
3	12.07	9.70	18	14.66	10.13
4	19.83	20.04	19	7.11	4.74
5	18.89	13.00	20	16.38	9.55
6	4.60	4.17	21	13.58	11.35
7	13.36	14.08	22	10.20	12.72
8	24.21	19.11	23	2.08	2.08
9	14.80	14.08	24	19.97	9.27
10	18.10	17.03	25	10.13	9.48
11	10.06	4.45	26	0.00	0.00
12	15.52	22.49	27	8.91	8.76
13	12.72	13.99	28	27.23	28.81
14	22.56	24.71	29	15.54	14.83
15	20.55	17.10	30	3.46	3.05
EER Features Fusion Result				13.17	12.42

**Table 5:** Showing authentication evaluation of success rate.

Data Authentication Performance						
Threshold (EER)	Patient			Non-Patient		
	Acceptance	Rejection	Success Rate	Acceptance	Rejection	Success Rate
9%	54	46	54%	0	2000	0%
10%	56	44	56%	0	2000	0%
11%	81	19	81%	6	1994	0.3%
12%	84	16	84%	8	1992	0.4%

## References

[1] https://www.statista.com/statistics/330695/number-of-smartphone-users-worldwide/.

[2] Khodadadi T., Islam A.M., Baharun S., Komaki S. (2016). Evaluation of recognition-based graphical password schemes in terms of usability and security attributes. Int. J. Electr. Comput. Eng..

[3] Saevanee H., Clarke N., Furnell S. SMS linguistic profiling authentication on mobile device. Proceedings of the 2011 5th IEEE International Conference on Network and System Security, Institute of Electrical and Electronics Engineers.

[4] Enamamu T.S., Clarke N., Dowland P.S., Li F. Smartwatch based body-temperature authentication. Proceedings of the IEEE International Conference on Computing Networking and Informatics (ICCNI).

[5] Peng Z., Chu F. (2004). Application of the wavelet transform in machine condition monitoring and fault diagnostics: A review with bibliography. Mech. Syst. Signal Process..

[6] Enamamu T.S., Clarke N., Dowland P.S., Li F. Transparent authentication: Utilising heart rate for user authentication. Proceedings of the 12th IEEE International Conference for Internet Technology and Secured Transactions (ICITST), Institute of Electrical and Electronics Engineers.

[7] https://www.who.int/goe/publications/goe_mhealth_web.pdf.

[8] AlMotiri S.H., Khan M.A., Alghamdi M.A. Mobile Health (m-Health) System in the Context of IoT. Proceedings of the IEEE 4th International Conference on Future Internet of Things and Cloud Workshops (FiCloudW).

[9] Mallat S.G., Heil C., Walnut D.F. (2009). A Theory for Multiresolution Signal Decomposition: The Wavelet Representation. IEEE Trans. Pattern Anal. Mach. Intell..

[10] Addison P.S., Walker J., Guido R.C. (2009). Time–frequency analysis of biosignals. IEEE Eng. Med. Biol. Mag..

[11] Subasi A., Ercelebi E. (2005). Classification of EEG signals using neural network and logistic regression. Comput. Methods Programs Biomed..

[12] Subasi A. (2007). EEG signal classification using wavelet feature extraction and a mixture of expert model. Expert Syst. Appl..

[13] Jahankhani P., Kodogiannis V., Revett K. EEG Signal Classification Using Wavelet Feature Extraction and Neural Networks. Proceedings of the IEEE John Vincent Atanasoff 2006 International Symposium on Modern Computing (JVA’06).

[14] Gokhale M.Y., Khanduja D.K. (2010). Time Domain Signal Analysis Using Wavelet Packet Decomposition Approach. Int. J. Commun. Netw. Syst. Sci..

[15] Prabhakar S., Mohanty A., Sekhar A. (2002). Application of discrete wavelet transform for detection of ball bearing race faults. Tribol. Int..

[16] Ovanesova A., Suarez L. (2004). Applications of wavelet transforms to damage detection in frame structures. Eng. Struct..

[17] Tsou C., Hsieh C.-H., Liang M.-C., Huang P.-W., Lee S.-Y. ECG acquisition system with heart rate detection and energy harvesting for drivers. Proceedings of the IEEE International Symposium on Bioelectronics and Bioinformatics (ISBB).

[18] Laine A., Fan J. (1993). Texture classification by wavelet packet signatures. IEEE Trans. Pattern Anal. Mach. Intell..

[19] Büssow R. (2007). An algorithm for the continuous Morlet wavelet transform. Mech. Syst. Signal Process..

[20] Li L., Liu P., Xing Y., Guo H. (2018). Time-frequency analysis of acoustic signals from a high-lift configuration with two wavelet functions. Appl. Acoust..

[21] Engelbrektsson J., Abrahamsson K., Breitholtz J., Nicholas M., Svensson O., Wikström H., Josefson M. (2010). The impact of Mexican hat and dual-tree complex wavelet transforms on multivariate evaluation of image texture properties. J. Chemom..

[22] Semwogerere D. (1998). The Use of the Two-Dimensional Continuous Wavelet Transform for Classification of Targets in Flir Imagery. Master’s Thesis.

[23] Sharif I., Khare S. (2014). Comparative Analysis of Haar and Daubechies Wavelet for Hyper Spectral Image Classification. ISPRS-Int. Arch. Photogramm. Remote. Sens. Spat. Inf. Sci..

[24] Mahmoodabadi S.Z., Ahmadian A., Abolhasani M.D. ECG feature extraction using Daubechies wavelets. Proceedings of the 5th IASTED International conference on Visualization, Imaging and Image Processing.

[25] Clonda D., Lina J.-M., Goulard B. (2004). Complex Daubechies wavelets: Properties and statistical image modelling. Signal Process..

[26] Misiti Y., Misiti M., Oppenheim G., Poggi J.-M. (1996). Wavelet Toolbox.

[27] Černá D., Finek V., Najzar K. (2008). On the exact values of coefficients of coiflets. Cent. Eur. J. Math..

[28] Stolojescu C., Railean I., Moga S., Isar A. Comparison of wavelet families with application to WiMAX traffic forecasting. Proceedings of the 12th IEEE International Conference on Optimization of Electrical and Electronic Equipment.

[29] Sindhura S.K., Reddy S.N., Kamaraj P. (2015). Comparison of SNR Improvement for Lower Atmospheric Signals Using Wavelets. Int. J. Adv. Res. Electr. Electron. Instrum. Eng..

[30] Abhyankar A., Schuckers S. (2010). Novel Biorthogonal Wavelet based Iris Recognition for Robust Biometric System. Int. J. Comput. Theory Eng..

[31] Hariprasath S., Venkatasubramaniam S. Iris pattern recognition using biorthogonal Wavelet Packet Analysis. Proceedings of the IEEE International Conference on Communication Control and Computing Technologies.

[32] Szewczyk R., Grabowski K., Napieralska M., Sankowski W., Zubert M., Napieralski A. (2012). A reliable iris recognition algorithm based on reverse biorthogonal wavelet transform. Pattern Recognit. Lett..

[33] Prashar D., Mnupreet K. (2014). Human Eye Iris Recognition Using Discrete 2d Reverse Biorthogonal Wavelet 6.8. Int. J. Sci. Technol. Res..

[34] Isnanto R.R. Iris recognition analysis using biorthogonal wavelets tranform for feature extraction. Proceedings of the 1st IEEE International Conference on Information Technology, Computer, and Electrical Engineering.

[35] Kapogiannopoulos G.S., Papadakis M. (1996). Character recognition using a biorthogonal discrete wavelet transform. SPIE’s Int. Symp. Optical Sci. Eng. Instrum..

[36] Terwiesch P., Mercorelli P. A local feature extraction using biorthogonal bases for classification of embedded classes of signals. Proceedings of the 14th International Symposium on Mathematical Theory of Networks and Systems.

[37] Hema C., Paulraj M., Kaur H. Brain signatures: A modality for biometric authentication. Proceedings of the IEEE International Conference on Electronic Design.

[38] Kousarrizi M.R.N., Ghanbari A.A., Teshnehlab M., Shoorehdeli M.A., Gharaviri A. Feature Extraction and Classification of EEG Signals Using Wavelet Transform, SVM and Artificial Neural Networks for Brain Computer Interfaces. Proceedings of the IEEE International Joint Conference on Bioinformatics, Systems Biology and Intelligent Computing.

[39] Guennoun M., Abbad N., Talom J., Rahman S.M.M., El-Khatib K. Continuous authentication by electrocardiogram data. Proceedings of the IEEE Toronto International Conference Science and Technology for Humanity (TIC-STH).

[40] Pirbhulal S., Zhang H., Mukhopadhyay S.C., Li C., Wang Y., Li G., Wu W., Zhang Y.-T. (2015). An Efficient Biometric-Based Algorithm Using Heart Rate Variability for Securing Body Sensor Networks. Sensors.

[41] Tawfik M.M., Kamal H.S.T. (2011). Human Identification Using QT Signal and QRS Complex of the ECG. OJEEE.

[42] Pourbabaee B., Howe-Patterson M., Reiher E., Benard F. (2018). Deep Convolutional Neural Network for ECG-Based Human Identification.

[43] Labati R.D., Munoz E., Piuri V., Sassi R., Scotti F. (2019). Deep-ECG: Convolutional Neural Networks for ECG biometric recognition. Pattern Recognit. Lett..

[44] Enamamu, Timibloudi S. (2019). Bioelectrical User Authentication. Ph.D. Thesis.

[45] Tay D.B.H., Marusic S., Deng G., Palaniswami M. (2004). Wavelet theoretic properties of the family of 6/10 biorthogonal filters. IEEE Int. Symp. Commun. Inf. Technol..

[46] Reaz M.B.I., Hussain M.S., Mohd-Yasin F., Raez M. (2006). Techniques of EMG signal analysis: Detection, processing, classification and applications (Correction). Biol. Proced. Online.

[47] Faris H., Aljarah I., Mirjalili S. (2016). Training feedforward neural networks using multi-verse optimizer for binary classification problems. Appl. Intell..

[48] Guez A., Selinsky J. (1989). Neurocontroller design via supervised and unsupervised learning. J. Intell. Robot. Syst..

[49] Fernández-Alemán J.L., Señor I.C., Lozoya P., Ángel O., Toval A. (2013). Security and privacy in electronic health records: A systematic literature review. J. Biomed. Inform..

